# 593. Randomized, Double-Blinded, Placebo-Controlled, Phase 1 Study of the Safety of BPL-1357, A BPL-Inactivated, Whole-Virus, Universal Influenza Vaccine

**DOI:** 10.1093/ofid/ofae631.188

**Published:** 2025-01-29

**Authors:** Luca Tudor Giurgea, Alison Han, Lindsay Czajkowski, Holly Ann Baus, Gregory Mak, Kimberly Adao, Ian Bellayr, Adriana Cervantes-Medina, Monica Gouzoulis, Jenna Sherry, Rani Athota, Viviane Callier, Sally Hunsberger, Jaekeun Park, Jeffery Taubenberger, Matthew J Memoli

**Affiliations:** National Institute of Allergy and Infectious Diseases, Bethesda, MD; National Institutes of Health, Bethesda, Maryland; National Institute of Allergy and Infectious Diseases, Bethesda, MD; National Institute of Allergy and Infectious Diseases, Bethesda, MD; LID Clinical Studies Unit, Laboratory of Infectious Diseases, Division of Intramural Research, National Institute of Allergy and Infectious Diseases, National Institutes of Health, Bethesda, Maryland; LID Clinical Studies Unit, Laboratory of Infectious Diseases, Division of Intramural Research, National Institute of Allergy and Infectious Diseases, National Institutes of Health, Bethesda, Maryland; Laboratory of Infectious Diseases, National Institute of Allergy and Infectious Diseases, National Institutes of Health, Bethesda, Maryland; National Institute of Allergy and Infectious Diseases, Bethesda, MD; LID Clinical Studies Unit, Laboratory of Infectious Diseases, Division of Intramural Research, National Institute of Allergy and Infectious Diseases, National Institutes of Health, Bethesda, Maryland; LID Clinical Studies Unit, Laboratory of Infectious Diseases, Division of Intramural Research, National Institute of Allergy and Infectious Diseases, National Institutes of Health, Bethesda, Maryland; National Institute of Allergy and Infectious Diseases, Bethesda, MD; Frederick National Laboratory for Cancer Research, Bethesda, Maryland; National Institute of Allergy and Infectious Diseases, Bethesda, MD; Department of Veterinary Medicine, VA-MD College of Veterinary Medicine, University of Maryland, College Park, Maryland; National Institute of Allergy and Infectious Diseases, Bethesda, MD; National Institute of Allergy and Infectious Diseases, Bethesda, MD

## Abstract

**Background:**

Better influenza vaccines are needed, not just for seasonal strains but for future pandemic viruses. BPL-1357 is a whole-virus, β-propiolactone-inactivated vaccine containing four wild-type, low-pathogenicity avian influenza viruses (H1N9, H3N8, H5N1, and H7N3). In mice and ferrets, BPL-1357 provided robust protection against challenge with a diverse set of influenza A viruses, including completely heterosubtypic strains.

**Methods:**

A phase 1, randomized, placebo-controlled, double-blinded, study was performed in volunteers 18 to 55 years of age using a single dose-level of vaccine given twice 28-days apart, either intramuscularly (IM) or intranasally (IN). Forty-five participants were block randomized (1:1:1) to receive IM BPL-1357 and IN placebo (IM group), IM placebo and IN BPL-1357 (IN group), or IM placebo and IN placebo (placebo group). Adverse events (AEs) were solicited using a questionnaire for 7 days after each vaccination and volunteers had medical follow-up for 182 days after the second vaccine (V_2_D182). Solicited AEs were presented visually as proportions. AEs were compared using tests of proportions at day 28 (V_2_D28) and V_2_D182.

**Results:**

Forty-five participants were enrolled between June 27, 2022 and November 9, 2022 and randomized into each group (n=15,15,15). Two participants (placebo group) delayed or missed their second dose because they developed COVID-19. No serious AEs were observed. Most AEs were mild. Injection site pain was the most common AE, which occurred in 87%/67% of the IM group after each vaccine dose respectively, but only in 33%/0% of the IN group, and 13%/21% of the placebo group. Other AEs, such as fatigue and headache, were similarly observed across the three groups. There was a severe AE possibly related to the IN vaccine (fever 39-40°C) which occurred after the first dose, but not the second. When comparing intervention-related AEs, only injection site pain was found to occur more frequently in the combined IM+IN group relative to placebo.

**Conclusion:**

BPL-1357 was safe and well-tolerated. Pain at the injection site was commonly seen in the IM group, though was mostly mild. Other AEs were similar in frequency between vaccine and placebo groups. Further work to determine the immunogenicity and efficacy of BPL-1357 is underway.

**Disclosures:**

**All Authors**: No reported disclosures

